# Fiske Medical Prize Questions

**Published:** 1857-09

**Authors:** 


*Fiske Medical Prize Questions.*

The Trustees of the Fiske Fund, at the annual meeting of the Rhode Island Medical Society, held at Providence, June 3, 1857, announced that the premium of one hundred dollars offered by them in 1857, for the best dissertation on the following subject:

"What are the causes and nature of that disease incident to pregnancy and lactation, characterized by inflammation and ulceration of the mouth and fauces, usually accompanied by anorexia, emaciation and diarrhœa, and what is the best mode of treatment?" has been awarded to the author of the dissertation bearing this motto:

"Wheat from the fields of science and cockles from my own farm."

And upon breaking the seal of the accompanying packet they learned that the successful competitor was David Hutchinson, M. D., of Mooresville, Morgan county, Indiana.

They propose the following subjects for 1858:

1st. The effects of the use of alcoholic liquors on tubercular disease, or in constitutions predisposed to such disease.

To be supported by facts presented as far as possible in a statistical form.

2d. The morbid effects of retention in the blood of the elements of the urinary secretions.

For the best dissertation on each of these subjects the Trustees will pay one hundred dollars.

Every competitor for a premium is expected to conform to the following regulations, viz:

To forward to the Secretary of the Trustees, on or before the first day of May, 1858, free of all expense, a copy of his dissertation, with a motto written thereupon, and also accompanying a sealed packet, having the same motto inscribed upon the outside, and his name and place of residence within.

Previously to receiving the premium awarded, the author of the successful dissertation must transfer to the Trustees all his right, title and interest in and to the same, for the use, benefit and behoof of the Fiske Fund.

Letters accompanying the unsuccessful dissertations will be destroyed by the Trustees, unopened, and the dissertations may be procured by their respective authors, if application be made therefor within three months.

ISAAC RAY, M. D., Providence,
JAS. H. ELDRIDGE, M. D., E. Greenwich, } Trustees.
CHAS. W. PARSONS, M. D., Providence,
S. AUG. ARNOLD, M. D., Providence, Sec'y.

